# 2-(3-Methyl­but-2-en-1-yl)-1,2-benziso­thia­zol-3(2*H*)-one 1,1-dioxide

**DOI:** 10.1107/S1600536809012021

**Published:** 2009-04-08

**Authors:** Muhammad Nadeem Arshad, M. Nawaz Tahir, Islam Ullah Khan, Muhammad Humayun Bilal, Hafiz Mubashar-ur-Rehman

**Affiliations:** a Department of Chemistry, Government College University, Lahore, Pakistan; b Department of Physics, University of Sargodha, Sargodha, Pakistan

## Abstract

In the title compound, C_12_H_13_NO_3_S, a saccharin derivative, the dihedral angle between the aromatic and isothia­zole rings is 2.91 (12)°. The planar 3,3-dimethyl­allyl group [maximum deviation = 0.0086 (16) Å] is oriented at dihedral angles of 71.86 (7) and 74.35 (7)° with respect to the aromatic and isothia­zole rings, respectively. In the crystal structure, weak inter­molecular C—H⋯O inter­actions link the mol­ecules into chains along the *c* axis. A weak C—H⋯π inter­action is also present.

## Related literature

For the biological activity of saccharine derivatives, see: Primofiore *et al.* (1997 ▶). For related structures, see: Arshad *et al.* (2008 ▶); Kruszynski & Czestkowski (2001 ▶); Siddiqui *et al.* (2007 ▶); Yu *et al.* (2008 ▶). For bond-length data, see: Allen *et al.* (1987 ▶).
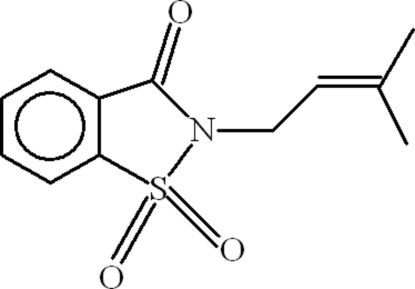


## Experimental

### 

#### Crystal data

C_12_H_13_NO_3_S
*M* *_r_* = 251.29
Orthorhombic, 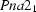

*a* = 9.4120 (5) Å
*b* = 19.4108 (11) Å
*c* = 6.5261 (4) Å
*V* = 1192.28 (12) Å^3^
*Z* = 4
Mo *K*α radiation
μ = 0.27 mm^−1^
*T* = 296 K
0.32 × 0.24 × 0.22 mm

#### Data collection

Bruker Kappa APEXII CCD area-detector diffractometer
Absorption correction: multi-scan (*SADABS*; Bruker, 2005 ▶) *T*_min_ = 0.924, *T*_max_ = 0.946
7340 measured reflections
2525 independent reflections
2304 reflections with *I* > 2σ(*I*)
*R*_int_ = 0.019

#### Refinement

*R*[*F* ^2^ > 2σ(*F* ^2^)] = 0.029
*wR*(*F* ^2^) = 0.083
*S* = 1.05
2525 reflections
156 parameters
1 restraint
H-atom parameters constrained
Δρ_max_ = 0.28 e Å^−3^
Δρ_min_ = −0.20 e Å^−3^
Absolute structure: Flack (1983 ▶), 837 Friedel pairs
Flack parameter: 0.02 (8)

### 

Data collection: *APEX2* (Bruker, 2007 ▶); cell refinement: *SAINT* (Bruker, 2007 ▶); data reduction: *SAINT*; program(s) used to solve structure: *SHELXS97* (Sheldrick, 2008 ▶); program(s) used to refine structure: *SHELXL97* (Sheldrick, 2008 ▶); molecular graphics: *ORTEP-3 for Windows* (Farrugia, 1997 ▶) and *PLATON* (Spek, 2009 ▶); software used to prepare material for publication: *WinGX* publication routines (Farrugia, 1999 ▶) and *PLATON*.

## Supplementary Material

Crystal structure: contains datablocks global, I. DOI: 10.1107/S1600536809012021/hk2656sup1.cif

Structure factors: contains datablocks I. DOI: 10.1107/S1600536809012021/hk2656Isup2.hkl

Additional supplementary materials:  crystallographic information; 3D view; checkCIF report

## Figures and Tables

**Table 1:** Hydrogen-bond geometry (Å, °)

*D*—H⋯*A*	*D*—H	H⋯*A*	*D*⋯*A*	*D*—H⋯*A*
C5—H5⋯O1^i^	0.93	2.56	3.391 (2)	149
C8—H8*A*⋯O2^ii^	0.97	2.51	3.436 (3)	160
C3—H3⋯*Cg*1^iii^	0.93	2.89	3.664 (2)	141

Symmetry codes: (i) 
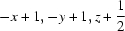
; (ii) 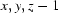; (iii) 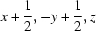. *Cg*1 is the centroid of the C1–C6 ring.

**Fig. 1.**
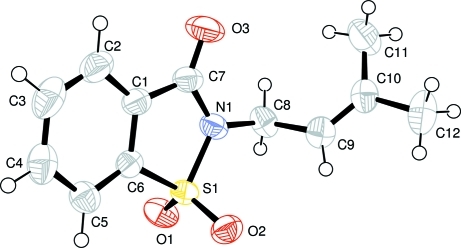
The molecular structure of the title molecule, with the atom-numbering scheme. Displacement ellipsoids are drawn at the 50% probability level.

**Fig. 2.**
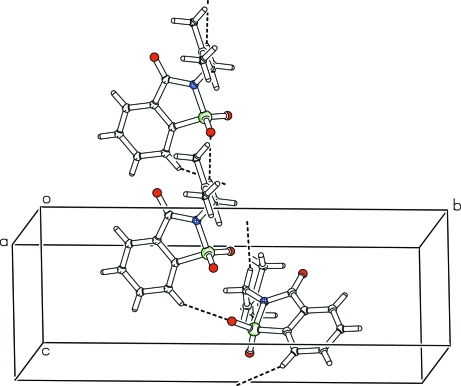
A partial packing diagram of the title compound. Hydrogen bonds are shown as dashed lines.

## supplementary crystallographic information

### Comment

The sodium salt of 1,2-benzisothiazole-3(2*H*)-one-1,1-dioxide is commonly known as saccharine, a sweetener. The derivatives of this compound are biologically active (Primofiore *et al.*, 1997) and used for the syntheses of various biologically active heterocyclic compounds. We report herein the crystal structure of the title compound, (I), as part of our ongoing studies on thiazine related heterocycles (Arshad *et al.*, 2008).

The crystal structures of 3-methylbut-2-enyl)ammonium chloride, (II) (Kruszynski & Czestkowski, 2001), 2-(chloromethyl)-1,2-benzisothiazole-1,1,3(2*H*) -trione, (III) (Siddiqui *et al.*, 2007) and 2-*n*-butyl-1,2-benziso- thiazol-3(2*H*)-one, (IV) (Yu *et al.*, 2008) have been published.

In the molecule of (I) (Fig 1), the bond lengths (Allen *et al.*, 1987) and angles are within normal ranges. Rings A (C1-C6) and B (S1/N1/C1/C6/C7) are, of course, planar and they are oriented at a dihedral angle of 2.91 (12)°. So, benzisothiazole ring system is nearly coplanar. The 3,3-dimethylallyl moiety C (C8-C12) is also planar with a maximum deviation of 0.0086 (16) Å for C10 atom, and it is oriented with respect to rings A and B at dihedral angles of A/C = 74.35 (7) and B/C = 71.86 (7) °. Atoms O1, O2 and O3 are 1.2007 (17), -1.2296 (19) and -0.0441 (27) Å away from the ring plane of B, respectively.

In the crystal structure, weak intermolecular C-H···O interactions (Table 1) link the molecules into chains along the c axis, in which they may be effective in the stabilization of the structure. There also exists a weak C—H···π interaction (Table 1).

### Experimental

For the preparation of the title compound, sodium salt of saccharine (1 g, 4.88 mmol) was dissolved in dimethylformamide (5 ml) in a round bottom flask (25 ml) equipped with condenser. Then, 3,3-dimethylallyl bromide (0.73 g, 4.88 mmol) was added to the solution and stirred at 353-373 K for 3 h. The progress of the reaction was observed by TLC. At completion of reaction, the mixture was poured on ice, precipitates obtained were filtered, washed with distilled water and dried. The residue was recrystalized in methanol to obtain the suitable crystals of the title compound.

### Refinement

H atoms were positioned geometrically, with C-H = 0.93, 0.97 and 0.96 Å for aromatic, methylene and methyl H, respectively, and constrained to ride on their parent atoms, with U_iso_(H) = xU_eq_(C), where x = 1.5 for methyl H and x = 1.2 for all other H atoms.

### Crystal data

**Table:**

C_12_H_13_NO_3_S	*F*(000) = 528
*M**_r_* = 251.29	*D*_x_ = 1.400 Mg m^−^^3^
Orthorhombic, *P**n**a*2_1_	Mo *K*α radiation, λ = 0.71073 Å
Hall symbol: P 2c -2n	Cell parameters from 2818 reflections
*a* = 9.4120 (5) Å	θ = 2.4–28.8°
*b* = 19.4108 (11) Å	µ = 0.27 mm^−^^1^
*c* = 6.5261 (4) Å	*T* = 296 K
*V* = 1192.28 (12) Å^3^	Rod, colorless
*Z* = 4	0.32 × 0.24 × 0.22 mm

### Data collection

**Table:**

Bruker Kappa APEXII CCD area-detector diffractometer	2525 independent reflections
Radiation source: fine-focus sealed tube	2304 reflections with *I* > 2σ(*I*)
graphite	*R*_int_ = 0.019
Detector resolution: 7.40 pixels mm^-1^	θ_max_ = 28.8°, θ_min_ = 2.4°
ω scans	*h* = −12→12
Absorption correction: multi-scan (*SADABS*; Bruker, 2005)	*k* = −26→26
*T*_min_ = 0.924, *T*_max_ = 0.946	*l* = −8→4
7340 measured reflections	

### Refinement

**Table:**

Refinement on *F*^2^	Secondary atom site location: difference Fourier map
Least-squares matrix: full	Hydrogen site location: inferred from neighbouring sites
*R*[*F*^2^ > 2σ(*F*^2^)] = 0.029	H-atom parameters constrained
*wR*(*F*^2^) = 0.083	*w* = 1/[σ^2^(*F*_o_^2^) + (0.05*P*)^2^ + 0.1276*P*] where *P* = (*F*_o_^2^ + 2*F*_c_^2^)/3
*S* = 1.05	(Δ/σ)_max_ < 0.001
2525 reflections	Δρ_max_ = 0.28 e Å^−^^3^
156 parameters	Δρ_min_ = −0.20 e Å^−^^3^
1 restraint	Absolute structure: Flack (1983), 837 Friedel pairs
Primary atom site location: structure-invariant direct methods	Flack parameter: 0.02 (8)

### Special details

**Table:**

Geometry. Bond distances, angles *etc*. have been calculated using the rounded fractional coordinates. All su's are estimated from the variances of the (full) variance-covariance matrix. The cell e.s.d.'s are taken into account in the estimation of distances, angles and torsion angles
Refinement. Refinement of *F*^2^ against ALL reflections. The weighted *R*-factor *wR* and goodness of fit *S* are based on *F*^2^, conventional *R*-factors *R* are based on *F*, with *F* set to zero for negative *F*^2^. The threshold expression of *F*^2^ > σ(*F*^2^) is used only for calculating *R*-factors(gt) *etc*. and is not relevant to the choice of reflections for refinement. *R*-factors based on *F*^2^ are statistically about twice as large as those based on *F*, and *R*- factors based on ALL data will be even larger.

### Fractional atomic coordinates and isotropic or equivalent isotropic displacement parameters (Å^2^)

**Table:**

	*x*	*y*	*z*	*U*_iso_*/*U*_eq_	
S1	0.39059 (4)	0.43722 (2)	0.22028 (8)	0.0360 (1)	
O1	0.47203 (15)	0.49780 (6)	0.1846 (2)	0.0527 (5)	
O2	0.27683 (15)	0.44174 (7)	0.3626 (3)	0.0501 (5)	
O3	0.34253 (18)	0.31180 (9)	−0.2062 (3)	0.0681 (6)	
N1	0.32910 (16)	0.40665 (8)	−0.0015 (3)	0.0420 (5)	
C1	0.47927 (19)	0.31849 (9)	0.1051 (3)	0.0430 (6)	
C2	0.5491 (2)	0.25556 (10)	0.1123 (4)	0.0571 (7)	
C3	0.6312 (2)	0.24143 (11)	0.2810 (5)	0.0643 (8)	
C4	0.6463 (2)	0.28766 (11)	0.4407 (4)	0.0582 (8)	
C5	0.5766 (2)	0.35100 (10)	0.4341 (4)	0.0474 (6)	
C6	0.49541 (17)	0.36438 (8)	0.2640 (3)	0.0377 (5)	
C7	0.37937 (19)	0.34237 (10)	−0.0545 (3)	0.0449 (6)	
C8	0.2217 (2)	0.44531 (10)	−0.1171 (4)	0.0480 (6)	
C9	0.0741 (2)	0.43125 (10)	−0.0398 (4)	0.0492 (7)	
C10	−0.0319 (2)	0.40469 (9)	−0.1434 (4)	0.0476 (6)	
C11	−0.0249 (3)	0.38285 (16)	−0.3615 (5)	0.0740 (10)	
C12	−0.1738 (2)	0.39260 (14)	−0.0418 (5)	0.0718 (9)	
H2	0.54056	0.22393	0.00600	0.0685*	
H3	0.67821	0.19935	0.28810	0.0770*	
H4	0.70306	0.27651	0.55253	0.0698*	
H5	0.58470	0.38275	0.54021	0.0569*	
H8A	0.22741	0.43275	−0.26069	0.0577*	
H8B	0.24147	0.49421	−0.10592	0.0577*	
H9	0.05601	0.44262	0.09618	0.0591*	
H11A	0.06668	0.39430	−0.41673	0.1109*	
H11B	−0.09740	0.40615	−0.43825	0.1109*	
H11C	−0.03930	0.33398	−0.37040	0.1109*	
H12A	−0.16911	0.40717	0.09857	0.1074*	
H12B	−0.19663	0.34444	−0.04742	0.1074*	
H12C	−0.24574	0.41849	−0.11203	0.1074*	

### Atomic displacement parameters (Å^2^)

**Table:**

	*U*^11^	*U*^22^	*U*^33^	*U*^12^	*U*^13^	*U*^23^
S1	0.0419 (2)	0.0326 (2)	0.0335 (2)	−0.0031 (1)	−0.0001 (2)	−0.0011 (2)
O1	0.0694 (8)	0.0384 (5)	0.0503 (10)	−0.0151 (5)	−0.0077 (8)	0.0034 (6)
O2	0.0521 (8)	0.0565 (8)	0.0418 (9)	0.0060 (5)	0.0063 (7)	−0.0040 (7)
O3	0.0786 (10)	0.0716 (10)	0.0540 (10)	−0.0120 (8)	−0.0050 (9)	−0.0261 (8)
N1	0.0438 (8)	0.0479 (8)	0.0343 (9)	−0.0056 (6)	−0.0020 (7)	−0.0034 (7)
C1	0.0392 (9)	0.0402 (8)	0.0495 (12)	−0.0075 (6)	0.0084 (8)	−0.0088 (8)
C2	0.0494 (11)	0.0444 (9)	0.0775 (17)	−0.0011 (8)	0.0111 (11)	−0.0155 (11)
C3	0.0501 (11)	0.0438 (9)	0.099 (2)	0.0072 (8)	0.0129 (12)	0.0057 (12)
C4	0.0462 (10)	0.0532 (11)	0.0752 (18)	0.0057 (8)	−0.0045 (11)	0.0117 (11)
C5	0.0475 (10)	0.0434 (9)	0.0513 (14)	−0.0043 (7)	−0.0044 (9)	0.0014 (9)
C6	0.0352 (7)	0.0336 (6)	0.0442 (12)	−0.0041 (5)	0.0046 (7)	−0.0010 (7)
C7	0.0436 (9)	0.0482 (9)	0.0430 (12)	−0.0128 (7)	0.0093 (8)	−0.0107 (9)
C8	0.0458 (10)	0.0566 (10)	0.0417 (12)	−0.0100 (8)	−0.0051 (9)	0.0101 (9)
C9	0.0486 (10)	0.0525 (10)	0.0466 (14)	0.0003 (8)	0.0024 (9)	0.0071 (9)
C10	0.0421 (10)	0.0398 (8)	0.0609 (14)	0.0017 (7)	−0.0025 (10)	0.0120 (9)
C11	0.0618 (14)	0.0871 (17)	0.0730 (19)	−0.0177 (12)	−0.0137 (13)	−0.0061 (15)
C12	0.0427 (11)	0.0706 (14)	0.102 (2)	0.0039 (9)	0.0049 (13)	0.0159 (14)

### Geometric parameters (Å, °)

**Table:**

S1—O1	1.4229 (13)	C10—C11	1.487 (4)
S1—O2	1.4201 (17)	C10—C12	1.510 (3)
S1—N1	1.6679 (19)	C2—H2	0.9300
S1—C6	1.7475 (16)	C3—H3	0.9300
O3—C7	1.205 (3)	C4—H4	0.9300
N1—C7	1.379 (2)	C5—H5	0.9300
N1—C8	1.468 (3)	C8—H8A	0.9700
C1—C2	1.388 (3)	C8—H8B	0.9700
C1—C6	1.376 (3)	C9—H9	0.9300
C1—C7	1.478 (3)	C11—H11A	0.9600
C2—C3	1.373 (4)	C11—H11B	0.9600
C3—C4	1.383 (4)	C11—H11C	0.9600
C4—C5	1.394 (3)	C12—H12A	0.9600
C5—C6	1.373 (3)	C12—H12B	0.9600
C8—C9	1.503 (3)	C12—H12C	0.9600
C9—C10	1.311 (3)		
			
O1···C5^i^	3.391 (2)	C11···H8A	2.6500
O1···C8^ii^	3.347 (2)	C12···H2^viii^	3.0500
O2···C9	3.253 (3)	H2···O3	2.8800
O2···C12^iii^	3.416 (3)	H2···C9^vii^	3.0400
O3···C5^iv^	3.308 (3)	H2···C10^vii^	2.7700
O1···H8B	2.8800	H2···C12^vii^	3.0500
O1···H5^i^	2.5600	H3···C1^vii^	3.0900
O2···H9	2.7100	H3···C7^vii^	3.0400
O2···H8A^v^	2.5100	H4···O3^x^	2.6700
O2···H12C^iii^	2.7300	H5···O1^ii^	2.5600
O2···H11A^v^	2.6100	H8A···O2^iv^	2.5100
O3···H2	2.8800	H8A···O3	2.6100
O3···H8A	2.6100	H8A···C11	2.6500
O3···H4^vi^	2.6700	H8A···H11A	1.9700
C3···C7^vii^	3.591 (3)	H8B···O1	2.8800
C5···O1^ii^	3.391 (2)	H9···O2	2.7100
C5···O3^v^	3.308 (3)	H9···H12A	2.2300
C7···C3^viii^	3.591 (3)	H11A···O2^iv^	2.6100
C8···O1^i^	3.347 (2)	H11A···C8	2.6300
C9···O2	3.253 (3)	H11A···H8A	1.9700
C12···O2^ix^	3.416 (3)	H11B···H12C	2.5600
C1···H3^viii^	3.0900	H11C···H12B	2.5800
C7···H3^viii^	3.0400	H12A···H9	2.2300
C8···H11A	2.6300	H12B···H11C	2.5800
C9···H2^viii^	3.0400	H12C···H11B	2.5600
C10···H2^viii^	2.7700	H12C···O2^ix^	2.7300
			
O1—S1—O2	117.53 (8)	C1—C2—H2	121.00
O1—S1—N1	109.81 (8)	C3—C2—H2	121.00
O1—S1—C6	113.03 (8)	C2—C3—H3	119.00
O2—S1—N1	109.14 (9)	C4—C3—H3	119.00
O2—S1—C6	111.65 (9)	C3—C4—H4	120.00
N1—S1—C6	92.85 (8)	C5—C4—H4	120.00
S1—N1—C7	114.88 (14)	C4—C5—H5	122.00
S1—N1—C8	120.21 (14)	C6—C5—H5	121.00
C7—N1—C8	124.76 (18)	N1—C8—H8A	109.00
C2—C1—C6	119.48 (18)	N1—C8—H8B	109.00
C2—C1—C7	126.96 (18)	C9—C8—H8A	109.00
C6—C1—C7	113.49 (16)	C9—C8—H8B	109.00
C1—C2—C3	118.0 (2)	H8A—C8—H8B	108.00
C2—C3—C4	122.2 (2)	C8—C9—H9	117.00
C3—C4—C5	120.1 (2)	C10—C9—H9	116.00
C4—C5—C6	117.0 (2)	C10—C11—H11A	109.00
S1—C6—C1	109.79 (14)	C10—C11—H11B	109.00
S1—C6—C5	126.83 (15)	C10—C11—H11C	109.00
C1—C6—C5	123.28 (16)	H11A—C11—H11B	109.00
O3—C7—N1	123.57 (19)	H11A—C11—H11C	109.00
O3—C7—C1	127.42 (18)	H11B—C11—H11C	109.00
N1—C7—C1	108.99 (16)	C10—C12—H12A	109.00
N1—C8—C9	111.79 (19)	C10—C12—H12B	109.00
C8—C9—C10	127.0 (2)	C10—C12—H12C	109.00
C9—C10—C11	124.9 (2)	H12A—C12—H12B	109.00
C9—C10—C12	120.5 (2)	H12A—C12—H12C	110.00
C11—C10—C12	114.6 (2)	H12B—C12—H12C	109.00
			
O1—S1—N1—C7	116.40 (14)	C6—C1—C2—C3	0.6 (3)
O1—S1—N1—C8	−67.88 (16)	C7—C1—C2—C3	−176.00 (19)
O2—S1—N1—C7	−113.44 (14)	C2—C1—C6—S1	−177.54 (15)
O2—S1—N1—C8	62.29 (17)	C2—C1—C6—C5	−0.9 (3)
C6—S1—N1—C7	0.66 (15)	C7—C1—C6—S1	−0.5 (2)
C6—S1—N1—C8	176.38 (15)	C7—C1—C6—C5	176.15 (17)
O1—S1—C6—C1	−113.04 (13)	C2—C1—C7—O3	−0.8 (3)
O1—S1—C6—C5	70.51 (19)	C2—C1—C7—N1	177.73 (19)
O2—S1—C6—C1	111.81 (14)	C6—C1—C7—O3	−177.6 (2)
O2—S1—C6—C5	−64.64 (19)	C6—C1—C7—N1	0.9 (2)
N1—S1—C6—C1	−0.09 (14)	C1—C2—C3—C4	−0.3 (3)
N1—S1—C6—C5	−176.54 (17)	C2—C3—C4—C5	0.2 (3)
S1—N1—C7—O3	177.61 (17)	C3—C4—C5—C6	−0.4 (3)
S1—N1—C7—C1	−1.0 (2)	C4—C5—C6—S1	176.81 (15)
C8—N1—C7—O3	2.1 (3)	C4—C5—C6—C1	0.8 (3)
C8—N1—C7—C1	−176.49 (17)	N1—C8—C9—C10	−119.5 (2)
S1—N1—C8—C9	−83.37 (19)	C8—C9—C10—C11	0.3 (3)
C7—N1—C8—C9	91.9 (2)	C8—C9—C10—C12	178.9 (2)

Symmetry codes: (i) −*x*+1, −*y*+1, *z*−1/2; (ii) −*x*+1, −*y*+1, *z*+1/2; (iii) −*x*, −*y*+1, *z*+1/2; (iv) *x*, *y*, *z*−1; (v) *x*, *y*, *z*+1; (vi) *x*−1/2, −*y*+1/2, *z*−1; (vii) *x*+1/2, −*y*+1/2, *z*; (viii) *x*−1/2, −*y*+1/2, *z*; (ix) −*x*, −*y*+1, *z*−1/2; (x) *x*+1/2, −*y*+1/2, *z*+1.

### Hydrogen-bond geometry (Å, °)

**Table:**

*D*—H···*A*	*D*—H	H···*A*	*D*···*A*	*D*—H···*A*
C5—H5···O1^ii^	0.93	2.56	3.391 (2)	149
C8—H8A···O2^iv^	0.97	2.51	3.436 (3)	160
C3—H3···Cg1^vii^	0.93	2.89	3.664 (2)	141

Symmetry codes: (ii) −*x*+1, −*y*+1, *z*+1/2; (iv) *x*, *y*, *z*−1; (vii) *x*+1/2, −*y*+1/2, *z*.
